# Effect of Pectin and Copper Modification on the Performance of Pd/ZnO Catalyst in Liquid-Phase Hydrogenation and Photocatalytic Hydrogen Evolution

**DOI:** 10.3390/molecules30183806

**Published:** 2025-09-18

**Authors:** Alima M. Kenzheyeva, Alima K. Zharmagambetova, Eldar T. Talgatov, Aigul T. Zamanbekova, Aigul I. Jumekeyeva, Assemgul S. Auyezkhanova, Zhannur K. Myltykbayeva, Atıf Koca

**Affiliations:** 1 Laboratory of Organic Catalysis, D.V. Sokolskiy Institute of Fuel, Catalysis, and Electrochemistry, Kunaev Str. 142, Almaty 050010, Kazakhstan; a.zharmagambetova@ifce.kz (A.K.Z.); a.zamanbekova@ifce.kz (A.T.Z.); a.dzhumekeeva@ifce.kz (A.I.J.); a.auezkhanova@ifce.kz (A.S.A.); 2 Faculty of Natural Sciences and Geography, Abai Kazakh National Pedagogical University, Almaty 050010, Kazakhstan; 3 Research Institute of New Chemical Technologies and Materials, Al-Farabi Kazakh National University, Almaty 050040, Kazakhstan; zhannur.myltykbaeva@kaznu.edu.kz; 4 Department of Chemical Engineering, Faculty of Engineering, Marmara University, Istanbul 34854, Türkiye; akoca@marmara.edu.tr

**Keywords:** Pd/ZnO catalyst, 2-hexyn-1-ol hydrogenation, photocatalytic hydrogen production, pectin, copper, catalyst modification, biopolymer-modified catalysts

## Abstract

This study investigates the influence of pectin and copper incorporation on the catalytic properties of Pd/ZnO catalysts in the liquid-phase hydrogenation of 2-hexyn-1-ol and photocatalytic hydrogen evolution. A series of monometallic Pd/ZnO catalysts with varying pectin contents (0–8.1 wt%) and bimetallic PdCu-Pec/ZnO catalysts with different Pd to Cu mass ratios (3:1, 1:1, 1:3) were synthesized via sequential adsorption of the polymer and metal ions onto ZnO. The catalysts were characterized using TGA, EDX, IR spectroscopy, XRD, TEM, UV–Vis DRS, and XPS. Characterization confirmed successful modification and changes in surface properties. Pectin modification improved the distribution of Pd nanoparticles on the surface of ZnO, resulting in the enhanced catalytic performance of Pd-Pec/ZnO in both hydrogenation and hydrogen evolution reactions compared to unmodified Pd/ZnO. In contrast, copper addition led to a deterioration of catalytic properties in both processes, likely due to the inhibited reduction of Pd caused by Pd–Cu interactions. Among the catalysts studied, Pd-Pec/ZnO with low pectin content (1.8 wt%) exhibited the highest activity in both reactions. The hydrogenation of 2-hexyn-1-ol to cis-2-hexen-1-ol proceeded with high selectivity (96%) at a rate (W_C≡C_) of 3.3 × 10^−6^ mol/s, and the catalyst retained its activity over 30 consecutive runs. In the photocatalytic hydrogen evolution reaction, the rate reached 1.11 mmol/(h·g_cat_) and the catalyst maintained ~94% of its initial activity after three consecutive runs. These findings demonstrate the potential of biopolymer-modified ZnO composites for the design of multifunctional catalysts combining hydrogenation and photocatalytic activity.

## 1. Introduction

Catalytic hydrogenation is one of the most important chemical transformations that find its practical application in many industrial processes, including the production of specialty chemicals from petroleum [1]. For example, various functional groups, such as –C≡C, –C=O, –NO_2_, –C≡N, –COOR, and –CONH_2_, can be selectively hydrogenated to their corresponding alkenes, alcohols, and amines, which are key intermediates for the fine chemical, polymer, agrochemical, and pharmaceutical industries [1,2]. Among these transformations, the semi-hydrogenation of acetylenic alcohols (e.g., 2-butyne-1,4-diol, 3-hexyn-1-ol, 2-hexyn-1-ol, 2-methyl-3-butyn-2-ol) to the corresponding olefinic alcohols is of particular interest due to their broad application as intermediates in the synthesis of vitamins, perfumes, pharmaceuticals, and agrochemicals [3,4].

Palladium-based heterogeneous catalysts are among the most effective systems for acetylenic compounds hydrogenation, owing to their excellent ability to activate hydrogen and unsaturated bonds [2]. However, it is still highly challenging to control the selective hydrogenation chemistry of the catalysts [5]. Selectivity depends on the electronic and geometric structure of active sites that can be tuned using different approaches, such as the selection of a suitable support material or the incorporation of other metals into Pd catalysts [2]. Chen X. et al. [6] report that Pd species can be uniformly immobilized on zinc oxide by adjusting the pH value to 5.9–6.3 forming a strongly charged surface of the support, thereby anchoring oppositely [PdCl_4_]^2−^ ions. The following high-temperature reduction (150–500 °C) of the resulting Pd/ZnO catalyst led to formation of intermetallic PdZn active sites exhibiting superior activity, unique selectivity, as well as outstanding stability in the continuous-flow semi-hydrogenation of alkynols to *cis*-enols.

In the review [7], the authors discussed the prospects of application of bimetallic PdCu nanoparticles as promising catalysts for a wide range of chemical and electrochemical reactions, including hydrogenation of organic compounds. Li J. et al. [8] have reported that the separation of continuous Pd atoms and modification of the Pd electronic state by Cu atoms suppressed β-hydride formation and alkene adsorption, contributing to the high selectivity of the developed PdCu@α-Al_2_O_3_ catalyst in semi-hydrogenation of different substituted alkynes. Another study [9] revealed the influence of the pH value during Pd deposition on the catalytic performance of Pd–CuO/SiO_2_ catalysts for the selective semi-hydrogenation of 2-methyl-3-butyn-2-ol (MBY). At a deposition pH of approximately 5, partial dissolution of CuO occurred, allowing for Cu^2+^ ions to co-reduce with Pd^2+^ using NaBH_4_. This process led to the formation of a sub-nanometer PdCu alloy. The resulting alloy modified the adsorption strength of MBY on the metal surface and altered the reaction kinetics of its semi-hydrogenation, resulting in high selectivity (up to 95%) toward the desired product, 2-methyl-3-buten-2-ol. Oberhauser W. et al. [10] demonstrated that PdCu-alloy nanoparticles, immobilized on stereocomplexed poly(lactic acid), selectively hydrogenated alkynols to alkenols (98 % at 96 % substrate conversion), whereas the corresponding Pd- and Cu-based catalysts showed high isomerization and over-hydrogenation activity.

Another highly effective strategy for tuning catalyst performance involves surface modification with polymeric or small organic ligands. Such modifiers can control nanoparticle size and morphology, alter the electronic structure of active sites, and influence substrate adsorption pathways [11,12,13]. In particular, biopolymers such as pectin, chitosan, and cellulose derivatives have gained significant attention due to their natural abundance, environmental friendliness, and functional group diversity. These macromolecules can act as stabilizers, structure-directing agents, or co-catalytic supports, enabling better control over reaction selectivity and stability [14]. In our prior work [15], ZnO-supported Pd and PdAg nanocatalysts modified with polysaccharides (2-hydroxyethyl cellulose, chitosan, and pectin) were tested in liquid-phase hydrogenation of 2-hexyn-1-ol under mild conditions (0.1 MPa, 40 °C). The obtained results show that the selectivity to *cis*-hexen-1-ol can be improved by modification of a Pd/ZnO catalyst with silver and suitable polymer.

Zinc oxide has also been widely utilized for photocatalytic water-splitting due to its excellent performance, low cost, non-toxicity, thermal stability, and chemical stability. However, its photocatalytic efficiency is often limited by poor visible-light absorption and the rapid recombination of photogenerated electron–hole pairs. Coupling ZnO with metal or metal oxide nanoparticles can reduce the bandgap, enhance charge separation, and suppress recombination, thereby improving performance in photocatalytic H_2_ production [16,17]. For instance, Park J.S. reported that the decoration of ZnO nanowires with Pd nanoparticles significantly enhanced their photocatalytic activity [18]. Similarly, Manzoor M.F. et al. demonstrated that doping ZnO with copper improved H_2_ evolution by reducing electron–hole recombination [19].

Therefore, this work is aimed to study the effect of modification of the Pd/ZnO with pectin and copper on behavior of the resulting composite catalysts in liquid-phase hydrogenation of 2-hexyn-1-ol and photocatalytic H_2_ evolution reaction. The effect of modification on the physicochemical properties of the catalysts was also investigated and discussed.

## 2. Results and Discussion

### 2.1. Characterization of the Catalysts

Mono- and bimetallic Pd/ZnO, Pd-Pec/ZnO, and PdCu-Pec/ZnO catalysts were prepared via an adsorption method. The amounts of the polymer and metal ions adsorbed on ZnO were assessed by determining their concentrations in the mother liquor after the sorption process. The pectin concentration was determined by measuring the viscosity of the mother liquor and referencing a previously established calibration curve, while the concentration of metal ions was determined using a photoelectric colorimetric (PEC) method.

Table 1 summarizes the results of pectin adsorption onto ZnO for the Pec/ZnO and Pd-Pec/ZnO composites. The data reveal distinct differences in the adsorption behavior between the two systems. In the Pec/ZnO system, the degree of pectin adsorption decreases from 100% to 59% as the amount of pectin introduced into the system increases from 18.5 to 92.5 mg. This trend indicates a saturation of available adsorption sites on the surface of ZnO, leading to a plateau in the pectin content within the final composite, which reaches a maximum of approximately 5.0 wt%. In contrast, the Pd-Pec/ZnO system exhibited an adsorption degree of 96–100%. This enhanced adsorption performance is likely related to the formation of polymer–metal complexes between pectin molecules and Pd^2+^ ions. The interaction between palladium and functional groups of pectin (such as carboxyl and hydroxyl groups) may promote partial crosslinking of the polymer chains, increasing their affinity for the surface of ZnO. Depending on the amount of pectin introduced, the calculated pectin content was found to be 1.8 wt%, 3.4 wt%, and 5.0 wt% for the Pec/ZnO composites and 1.8 wt%, 3.5 wt%, and 8.1 wt% for the Pd-Pec/ZnO composites. For simplicity, the obtained Pec/ZnO and Pd-Pec/ZnO composites were designated as Pec1.8/ZnO, Pec3.4/ZnO, Pec5.0/ZnO, Pd-Pec1.8/ZnO, Pd-Pec3.5/ZnO, and Pd-Pec8.1/ZnO, where the number indicates the pectin content (in wt%) in the composite.

**Figure 1:**
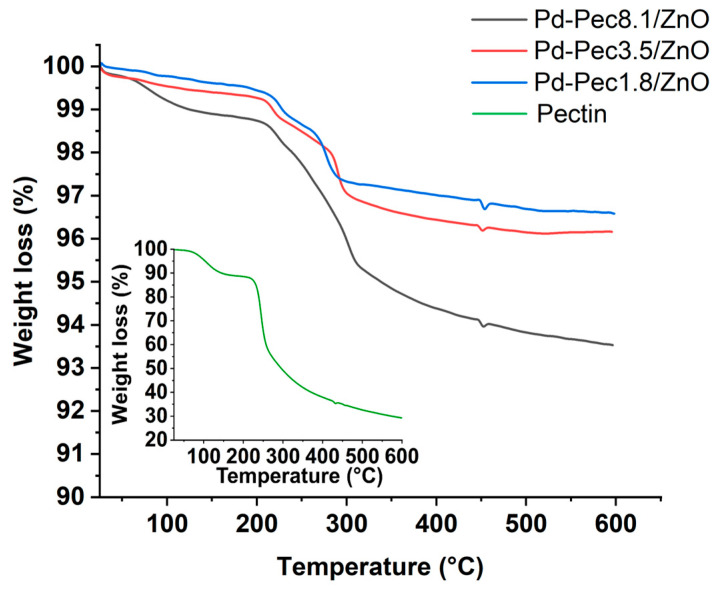
TGA of Pd-Pec1.8/ZnO, Pd-Pec3.5/ZnO, Pd-Pec8.1/ZnO and pectin (inset).

The presence and varying content of pectin in the composites was confirmed using thermogravimetric analysis (TGA) and infrared spectroscopy (IR). According to TGA data (Figure 1), the thermal degradation of pectin and Pd-Pec/ZnO composites occurred in two main stages. In the low-temperature region (up to ~220–280 °C), minor weight loss was observed, attributed to the evaporation of physically adsorbed water and the removal of other small molecules [20,21]. The main degradation step, associated with the decomposition of the pectin, occurs at higher temperatures. The variations in thermogram profiles confirm differences in the polymer content and structure across the samples [21,22].

The inset in Figure 1 shows the TGA curve of pure pectin, which exhibits a ~12.4% weight loss below 220 °C due to moisture removal. This is followed by a major weight loss (~55%) in the 220–500 °C range, corresponding to the thermal decomposition of the polymer backbone. It should be noted that the residual mass of ~33% for pure pectin at 500 °C is consistent with the values in the literature reported for commercial pectins (32–34%) [21,22]. Furthermore, the observed weight loss in the 220–500 °C range (~55%) closely matches previous data for apple pectin (~50%) [23].

In the Pd-Pec1.8/ZnO composite, pectin decomposition began at a higher temperature (~280 °C), indicating enhanced thermal stability. A weight loss of 2.2% below this temperature is attributed to the evaporation of physically bound water. In the 280–500 °C range, the weight loss was 1.1%. Given that pure pectin loses approximately 55% of its mass at this stage (Figure 1, inset), the pectin content in Pd-Pec1.8/ZnO was estimated to be around 2.0 wt%. This estimate is consistent with the viscosimetry data presented in Table 1, confirming the effective and quantitative deposition of pectin on the ZnO surface.

For the Pd-Pec3.5/ZnO composite, decomposition began at approximately 265 °C. A weight loss of 1.7% observed below this temperature is attributed to the evaporation of bound water. In the 265–500 °C range, the sample lost an additional 2.1% of its mass. Based on the weight loss characteristic of pure pectin, the calculated pectin content in the composite was approximately 3.8 wt%, which closely matched the viscosimetry-based result of 3.5 wt% (Table 1).

In the case of Pd-Pec8.1/ZnO, thermal decomposition of the polymer started at approximately 240 °C. A weight loss of 2.0% below this temperature is attributed to the release of bound water. A further 4.2% weight loss observed in the range of 240–500 °C indicated a pectin content of approximately 7.6 wt%, which is in good agreement with the value of 8.1 wt% obtained from viscosimetry (Table 1).

Importantly, the onset temperature of pectin decomposition was found to be inversely proportional to its content in the composite: 280 °C for Pd-Pec1.8/ZnO, 265 °C for Pd-Pec3.5/ZnO, 240 °C for Pd-Pec8.1/ZnO, and 220 °C for pure pectin. This shift can be attributed to differences in the degree of crosslinking between pectin and palladium ions. Since the Pd content remained constant across all samples, the Pd:pectin molar ratio varied, resulting in different levels of crosslinking. In Pd-Pec1.8/ZnO, the Pd:pectin molar ratio was approximately 1:1, indicating nearly complete crosslinking. In Pd-Pec3.5/ZnO and Pd-Pec8.1/ZnO, the ratios were approximately 1:2 and 1:5, respectively, corresponding to lower degrees of crosslinking and, consequently, reduced thermal stability. This interpretation is consistent with the literature data. For example, a pectin-based hydrogel (pectin-co-poly(MAA)) exhibited a significantly higher decomposition onset temperature (>400 °C) compared to pure pectin (~240 °C) [22]. Similarly, pectin derivatives, such as polyphenol-conjugated pectins, demonstrated enhanced structural stability, with decomposition starting at 240–250 °C, whereas native pectin began to decompose at approximately 230 °C [21].

**Figure 2:**
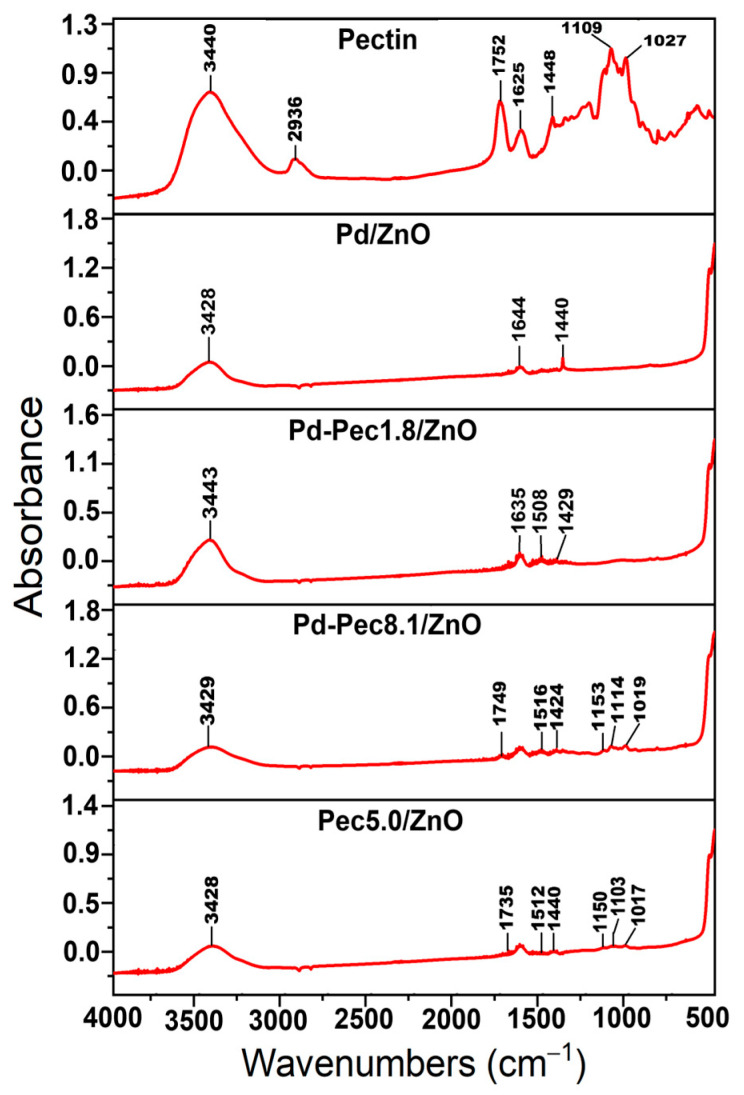
IR spectra of pectin, Pd/ZnO, Pd-Pec1.8/ZnO, Pd-Pec8.1/ZnO and Pec5.0/ZnO.

Figure 2 shows IR spectra of pectin, Pd/ZnO, Pd-Pec1.8/ZnO, Pd-Pec8.1/ZnO, and Pec5.0/ZnO. The IR spectrum of pectin displays characteristic absorption bands corresponding to various functional groups of the polymer. Specifically, the bands at 3440 cm^−1^, 1752 cm^−1^, and 1625 cm^−1^ are attributed to the stretching vibrations of –OH, C=O of ester and C=O of carboxylic acid groups, respectively [24]. Notably, the absorbance is higher at 1752 cm^−1^ than at 1625 cm^−1^, confirming a high degree of esterification of the pectin (more than 50%). In addition, the region between 1200 and 1500 cm^−1^ contains absorption bands associated with the stretching and bending vibrations of –C–OH, –C–H, and –O–H functional groups [25]. The broad absorption band in the range of 950–1200 cm^−1^ corresponds to the “fingerprint” region of carbohydrates, which is crucial for identifying key structural units such as C–O, C–O–C, and C–C bonds in polysaccharides [26]. The IR spectrum of Pd/ZnO exhibits characteristic bands at 400–500 cm^−1^, 1635 cm^−1^, and 3443 cm^−1^. The intensive band in the range of 400–500 cm^−1^ corresponds to the Zn–O stretching vibrations, which are typical for ZnO-based materials [27]. The band at 1635 cm^−1^ is assigned to the bending vibrations of adsorbed water molecules (H–O–H), while the broad absorption band at 3443 cm^−1^ is attributed to the O–H stretching vibrations of surface hydroxyl groups or physically adsorbed water [28]. The IR spectra of Pec5.0/ZnO and Pd-Pec/ZnO composites display absorption bands characteristic of both pectin and Pd/ZnO, confirming the successful incorporation of the polymer into the composite structure. Notably, the intensity of pectin-related bands (especially evident in the fingerprint region around 950–1200 cm^−1^) increases in the following order, Pd-Pec1.8/ZnO < Pec5.0/ZnO < Pd-Pec8.1/ZnO, reflecting the increasing pectin content in the composites. This trend is consistent with the results obtained from viscosimetry (Table 1) and TGA (Figure 1), further confirming the varying amounts of polymer deposited on the ZnO surface. Moreover, a comparison of IR spectra of pectin, Pec5.0/ZnO, and Pd-Pec8.1/ZnO composites indicated interaction of pectin with both ZnO and Pd ions. This is evidenced by the shift of the ester carbonyl (COOR) band from 1752 cm^−1^ in pure pectin to 1735 cm^−1^ in Pec5.0/ZnO and 1749 cm^−1^ in Pd-Pec8.1/ZnO. Furthermore, new bands appearing at 1512 (Pec5.0/ZnO) and 1516 cm^−1^ (Pd-Pec8.1/ZnO) in the composite samples can be attributed to the asymmetric stretching vibrations of carboxylate anions (COO^−^) [29], indicating coordination between deprotonated carboxyl groups and Zn^2+^ or Pd^2+^ ions. Minor shifts in the fingerprint region (950–1200 cm^−1^) further support the structural modification of pectin upon interaction with the inorganic components.

According to photoelectric colorimetric (PEC) analysis data, palladium ions were quantitatively adsorbed (~100%) on ZnO, which can be explained by the hydrolysis of Pd ions in the presence of ZnO. Pec/ZnO composites with a low pectin content (up to ~3.5 wt%) also demonstrated high sorption capacity (96–99%) toward Pd^2+^ ions. In these cases, palladium ions likely interact with both ZnO and the pectin adsorbed on its surface. However, when the pectin content in the Pec/ZnO composite was increased to ~8 wt%, the degree of Pd adsorption decreased to 86.6%. This could be attributed to the binding of Pd^2+^ ions with excess pectin that was not adsorbed onto the ZnO surface. This assumption was further supported by an additional experiment: the clarification of the mother liquor upon ethanol addition to the Pd^2+^–Pec–ZnO system suggested the complete removal of the free Pd–Pec complex via precipitation. During the preparation of bimetallic catalysts, palladium was introduced into the system after copper. In these cases, the amount of palladium introduced was lower, which likely facilitated its more complete sorption. As a result, the degree of Pd^2+^ adsorption reached 99–100%. Copper ions were also effectively adsorbed in the PdCu–Pec1.8/ZnO composites, with adsorption degrees ranging from 90% to 96% depending on the Pd:Cu ratio. Based on PEC analysis, the total metal (Pd and Cu) content in the catalysts was approximately 1 wt%, which was consistent with the results obtained by energy-dispersive X-ray (EDX) elemental analysis (Table 2).

**Figure 3:**
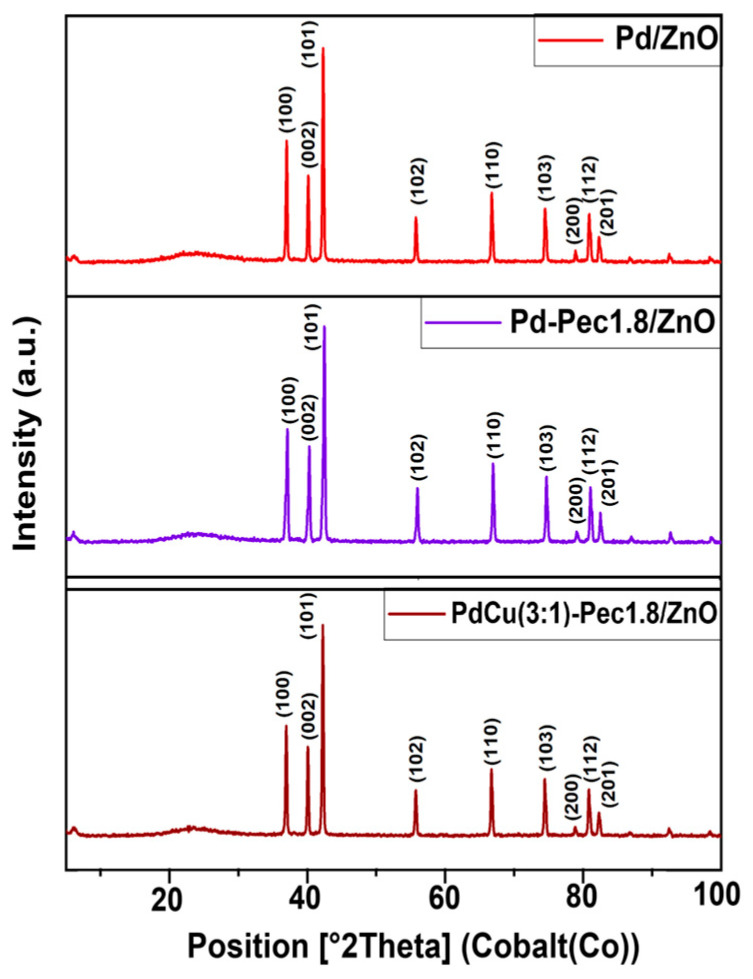
XRD patterns of Pd/ZnO, Pd-Pec1.8/ZnO, and PdCu(3:1)-Pec1.8/ZnO.

The results of X-ray powder diffraction analysis (XRD) of the Pd/ZnO, Pd-Pec1.8/ZnO, and PdCu(3:1)-Pec1.8/ZnO are shown in Figure 3. All XRD patterns showed characteristic peaks at 36.9°, 40.0°, 42.2°, 55.6°, 66.7°, 74.4°, 78.8°, 80.8°, and 82.3°, corresponding to the (100), (002), (101), (102), (110), (103), (200), (112), and (201) planes of ZnO hexagonal wurtzite structures (JCPDS card no. 79-2205) [30]. No diffraction peaks corresponding to palladium (Pd or PdO) or copper (Cu, Cu_2_O, or CuO) species were observed in the XRD patterns of the catalysts. This absence can be attributed to the low metal content (Pd and Cu) in the samples, as well as the small size and high dispersion of the metal particles [15].

**Figure 4:**
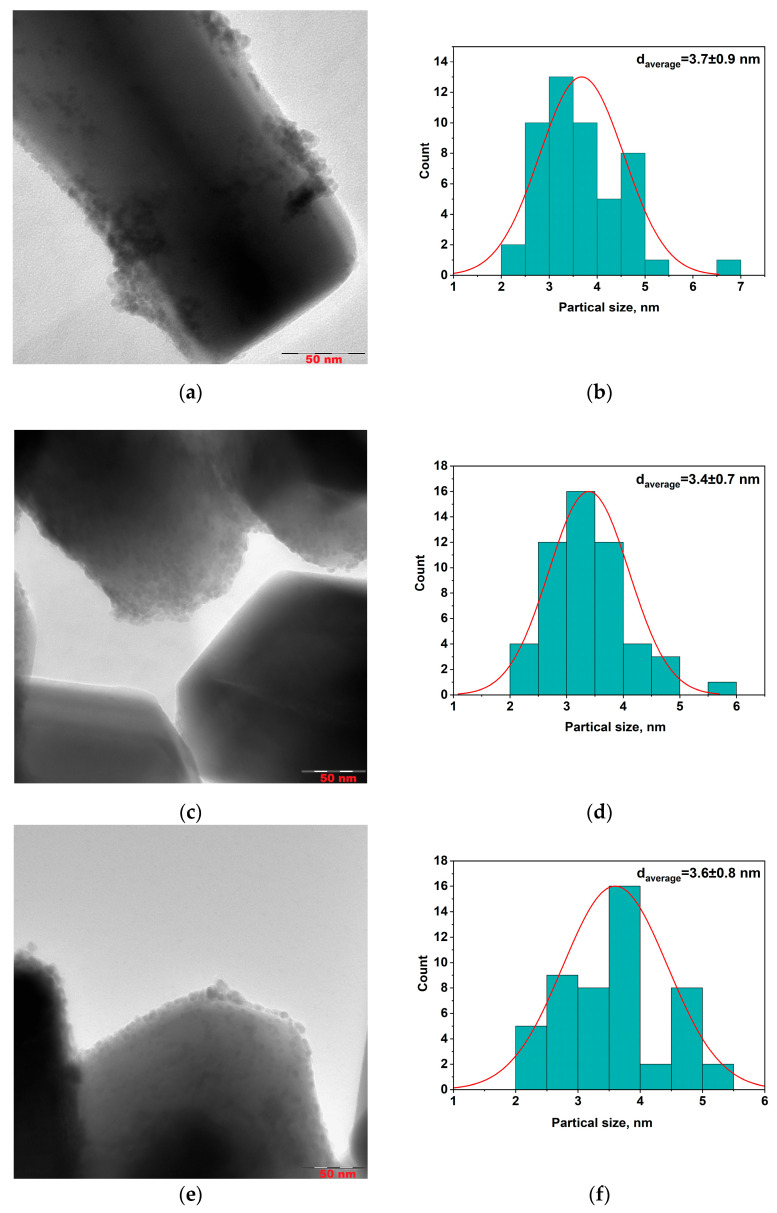
TEM microphotographs (**a**,**c**,**e**) and corresponding Pd and PdCu particle size distribution histograms (**b**,**d**,**f**) for Pd/ZnO (**a**,**b**), Pd-Pec1.8/ZnO (**c**,**d**), and PdCu(3:1)-Pec1.8/ZnO (**e**,**f**).

To evaluate the size of metal particles and their distribution of a support surface Pd/ZnO, Pd-Pec1.8/ZnO, and PdCu(3:1)-Pec1.8/ZnO were studied using transmission electron microscopy (TEM) method (Figure 4). Prior to conducting the studies, the catalysts were treated with hydrogen (1 atm) in a reactor at 40 °C for 30 min.

**Figure 5:**
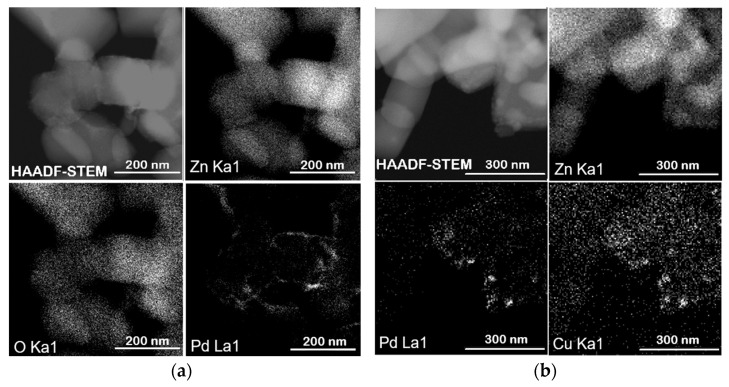
HAADF-STEM images with corresponding EDX elemental maps of Zn, O, Pd and Cu for Pd-Pec1.8/ZnO (**a**) and PdCu(3:1)-Pec1.8/ZnO (**b**).

According to TEM studies, the Pd/ZnO catalyst is composed of palladium (Pd) nanoparticles with a primary size of approximately 3.7 nm (Figure 4b). These nanoparticles tend to aggregate, forming larger nanoclusters ranging from 10 to 30 nm in size, which are distributed across various sites of the ZnO support. However, in certain regions, Pd nanoparticles remain well-dispersed without forming aggregates (Figure 4a). In contrast, both the Pd–Pec1.8/ZnO (Figure 4c) and bimetallic PdCu(3:1)–Pec1.8/ZnO (Figure 4e) catalysts exhibit a uniform distribution of nanoparticles on the ZnO support, with an average particle size of approximately 3.4–3.6 nm (Figure 4d,f). The nanoparticles are predominantly spherical or slightly oval in shape, and show no signs of significant aggregation compared to those of unmodified Pd/ZnO (Figure 4a,c,e). Additional high-magnification TEM images of the same catalysts are presented in the Supplementary Information (Figure S1a–c), confirming both the particle sizes indicated by the histograms (Figure 4b,d,f) and the particle morphology and dispersion. The use of pectin as a stabilizing agent during synthesis likely contributes to this homogeneous dispersion by preventing nanoparticle growth and aggregation. Elemental mapping analysis of the Pd–Pec1.8/ZnO catalyst (Figure 5a) further confirms the homogeneous distribution of palladium on the Pec1.8/ZnO support surface. The HAADF-STEM image, along with the corresponding EDX elemental maps for Zn (Zn Kα1), O (O Kα1), and Pd (Pd Lα1), shows that palladium is well dispersed across the support, without evidence of significant aggregation or phase separation. Similarly, the elemental maps of the PdCu(3:1)–Pec1.8/ZnO catalyst (Figure 5b) show uniform distribution and co-localization of Pd and Cu nanoparticles on the ZnO surface. The overlapping bright spots in the Pd and Cu maps suggest that both metals may be spatially coincident, which could indicate the formation of bimetallic nanoparticles or alloys.

**Figure 6:**
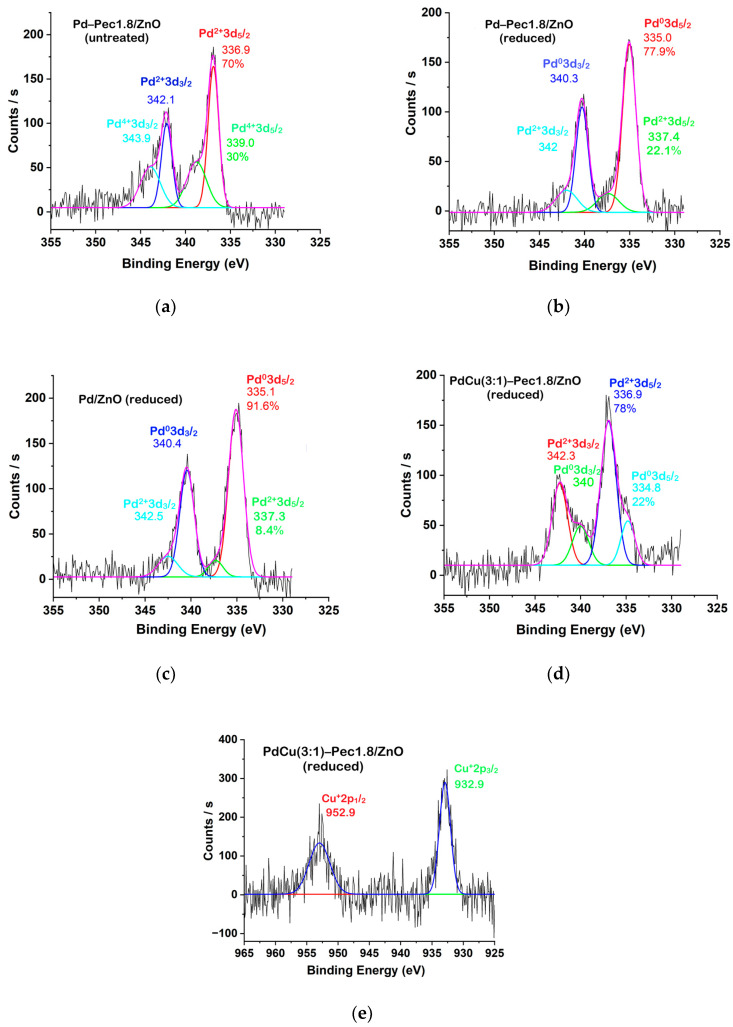
XPS spectra of Pd 3d (**a**–**d**) and Cu 2p (**e**) for untreated Pd-Pec1.8/ZnO (**a**), reduced Pd-Pec1.8/ZnO (**b**), reduced Pd/ZnO (**c**), and reduced PdCu(3:1)-Pec1.8/ZnO (**d**,**e**).

To assess the oxidation states of palladium and copper species during the hydrogenation process, X-ray photoelectron spectroscopy (XPS) was employed. The catalysts (Pd/ZnO, Pd-Pec1.8/ZnO, and PdCu(3:1)-Pec1.8/ZnO) were pretreated with molecular hydrogen at 40 °C in a reactor prior to XPS analysis. Additionally, untreated Pd-Pec1.8/ZnO was analyzed to determine the effect of hydrogen pretreatment on the Pd species. Figure 6 shows the Pd 3d and Cu 2p regions of the XPS spectra of the catalysts. The deconvoluted Pd 3d signals (Figure 6a–d) clearly illustrate the different oxidation states of Pd existing on the surface of the catalysts. In the untreated Pd-Pec1.8/ZnO, Pd 3d_5/2_ peaks with binding energies at 336.9 eV and 339.0 eV can be attributed to Pd in +2 and +4 oxidation states, respectively (Figure 6a) [31,32]. Treatment of the catalyst with molecular hydrogen at 40 °C led to significant changes in the Pd 3d spectral profile (Figure 6b). The peak at 339.0 eV (Pd^4+^) disappeared, and a new peak emerged at 335.0 eV, characteristic of metallic palladium (Pd^0^) [33,34]. In the reduced Pd-Pec1.8/ZnO sample, Pd^0^ (335.0 eV) became the dominant species, representing 78% of the surface Pd, while Pd^2+^ (337.4 eV) accounted for 22%. This confirms that hydrogen treatment effectively converts oxidized Pd species (Pd^4+^ and Pd^2+^) into metallic Pd^0^, thereby modulating the electronic state of palladium. Such modulation is expected to strongly influence catalytic behavior in hydrogenation reactions. Similarly, the reduced Pd/ZnO catalyst exhibited Pd 3d_5_/_2_ peaks at 335.1 eV and 337.3 eV, associated with Pd^0^ (92%) and Pd^2+^ (8%), respectively (Figure 6c). This suggests that the incorporation of pectin did not significantly affect the redox state of palladium after hydrogen treatment. In contrast, modification with copper led to notable changes in the Pd 3d spectral profile. The reduced PdCu(3:1)-Pec1.8/ZnO catalyst exhibited Pd_5_/_2_ peaks at 334.8 eV and 336.9 eV, with Pd^2+^ becoming the predominant species (78%) and Pd^0^ contributing only 22% (Figure 6d).

The Cu 2p_3_/_2_ region of the XPS spectrum (Figure 6e) displays a main peak at ~933 eV with a full width at half maximum (FWHM) of 2.1 eV, which is characteristic of Cu^+^ in Cu_2_O [35]. This suggests that Cu^2+^ species were effectively reduced to Cu^+^ during H_2_ pretreatment.

These observations suggest the formation of a core–shell-like structure, in which palladium forms the core and copper is preferentially located at the outer surface. This assumption is based on several experimental observations. The negative shift of the Pd 3d_5_/_2_ peak by 0.2 eV (from 335.0 eV in Pd-Pec1.8/ZnO to 334.8 eV in PdCu(3:1)-Pec1.8/ZnO) indicates electron transfer from Cu to Pd, in agreement with literature reports on PdCu systems [36,37]. This shift confirms that Pd and Cu are in direct contact and interact electronically. However, although electron donation from Cu to Pd would typically be expected to facilitate the reduction of Pd^2+^ to Pd^0^ [38], our data reveal the opposite trend: only 22% of Pd is reduced in the bimetallic system, compared to 78% in the monometallic analog. This apparent paradox can be reasonably explained by the physical blockage of Pd active sites by surface Cu atoms, which hinders hydrogen access and thus suppresses Pd reduction. In contrast, Cu^2+^ is fully reduced to Cu^+^, indicating that Cu is more surface-exposed, while Pd is likely more internally located, supporting a core–shell-like structural arrangement, in which Pd forms the core and Cu the shell. The enhanced reducibility of Cu is consistent with a previous report [39], where Pd facilitates Cu reduction through hydrogen spillover or synergistic electronic effects. Although this study does not directly confirm a core–shell structure, it supports the observed preferential reduction of Cu in bimetallic systems. Importantly, this assumption does not contradict the elemental mapping (EDX) data (Figure 5b), which show colocalization of Pd and Cu, but with a slightly broader spatial distribution of Cu. This observation may reflect the surface enrichment of Cu, and is compatible with a core–shell-like architecture where Pd resides predominantly in the particle interior. Taken together, these results indicate that Cu not only alters the electronic structure of Pd, but also modulates its accessibility to reducing agents, ultimately impacting the reducibility and catalytic performance of the bimetallic system.

### 2.2. Catalytic Properties of Pd and PdCu Catalysts in Hydrogenation Process

**Figure 7:**
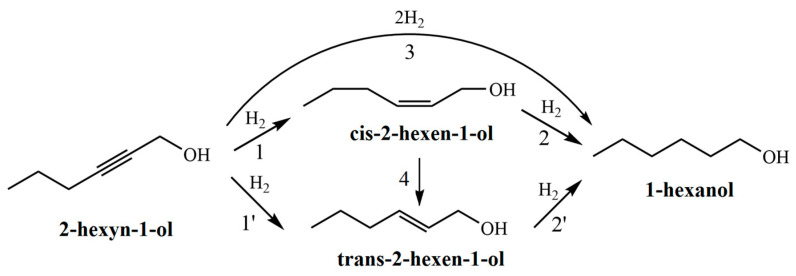
Plausible reaction pathways for the hydrogenation of 2-hexyn-1-ol. 1 and 1′—hydrogenation of the triple C≡C bond in 2-hexyn-1-ol to form cis-2-hexen-1-ol and trans-2-hexen-1-ol, respectively. 2 and 2′—hydrogenation of the C=C bonds in cis-2-hexen-1-ol and trans-2-hexen-1-ol, respectively, yielding 1-hexanol. 3—direct hydrogenation of the triple bond in 2-hexyn-1-ol to 1-hexanol. 4—isomerization of cis-2-hexen-1-ol to trans-2-hexen-1-ol.

The hydrogenation of 2-hexyn-1-ol proceeds via a consecutive reaction pathway (Figure 7). In the first step, the triple C≡C bond of the acetylenic alcohol is selectively reduced to a double C=C bond, forming both cis- and trans-isomers of 2-hexen-1-ol (Figure 7, reactions 1 and 1′). These intermediates are further hydrogenated to 1-hexanol (Figure 7, reactions 2 and 2′). Additionally, direct hydrogenation of 2-hexyn-1-ol to 1-hexanol without accumulation of intermediates is also possible (Figure 7, reaction 3). It should also be noted that cis-2-hexen-1-ol may isomerize to its trans counterpart under the reaction conditions (Figure 7, reaction 4).

To investigate the catalytic performance in these transformations, two sets of catalysts were studied. In the first part, a series of monometallic Pd/ZnO catalysts with varying pectin content were examined to evaluate the effect of pectin modification on activity and selectivity. In the second part, the performance of the optimized monometallic catalyst was compared with bimetallic PdCu-Pec/ZnO catalysts, in which the Pd to Cu mass ratio was systematically varied to assess the influence of copper incorporation on the hydrogenation behavior.

The activity of the catalysts was evaluated by measuring the hydrogen uptake as a function of time. Based on the H_2_ consumption data, the reaction rate was calculated. The selectivity of the process was determined by chromatographic analysis of reaction mixtures sampled at different time intervals.

**Figure 8:**
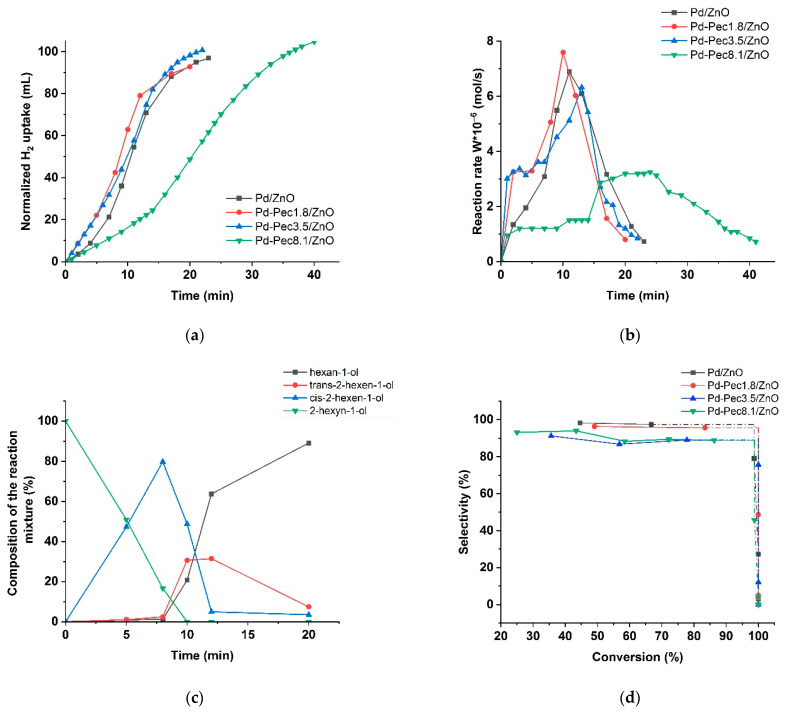
Hydrogenation of 2-hexyn-1-ol on Pd/ZnO and Pd-Pec/ZnO catalysts: hydrogen uptake (**a**); change in the rate of reaction (**b**); changes in the composition of the reaction mixture in presence of Pd-Pec/1.8ZnO (**c**); and dependence of selectivity to cis-2-hexen-1-ol with the substrate conversion (**d**). Reaction conditions: 50 mg of catalyst, 0.25 mL of 2-hexyn-1-ol, 25 mL of ethanol, at 40 °C and 0.1 MPa.

Figure 8 shows the results of testing the monometallic Pd/ZnO catalysts in the hydrogenation of 2-hexyn-1-ol. Figure 8a presents the variation in H_2_ uptake over time during the reaction. The Pd/ZnO and Pd-Pec/ZnO catalysts with lower pectin content demonstrated higher catalytic activity compared to Pd-Pec8.1/ZnO. The semi-hydrogenation point (50 mL of H_2_ uptake) was reached after 8, 9, 10, and 20 min for Pd-Pec1.8/ZnO, Pd-Pec3.5/ZnO, Pd/ZnO, and Pd-Pec8.1/ZnO, respectively. These results suggest that modification with a small amount of pectin slightly improves the activity of the Pd/ZnO catalyst. However, a further increase in pectin content leads to a decrease in activity, most likely due to the steric hindrance caused by the excess polymer, which limits substrate access to the Pd active sites [40]. Figure 8b shows the hydrogenation rate (W), calculated from the H_2_ uptake data. For all Pd-Pec/ZnO catalysts, the reaction rate increased during the first two minutes and remained nearly constant until the semi-hydrogenation point. Then, the rate increased again, reached a maximum, and subsequently dropped sharply. In contrast, the unmodified Pd/ZnO catalyst exhibited a slower and more gradual increase in reaction rate during the initial period up to the semi-hydrogenation point, without the sharp initial rise characteristic of the pectin-modified samples. This behavior can be attributed to a more uniform distribution of palladium particles on the support surface in the pectin-modified catalyst compared to the polymer-free system, as evidenced by the TEM data (Figure 4).

According to chromatographic analysis, cis-2-hexen-1-ol accumulated in the reaction medium over Pd-Pec1.8/ZnO during the initial period and was subsequently reduced to 1-hexanol (Figure 8c), which confirmed the occurrence of Paths 1 and 2 in Figure 7, respectively. The accumulation of cis-2-hexen-1-ol was accompanied by the formation of minor amounts of trans-2-hexen-1-ol and 1-hexanol, whose yields at the semi-hydrogenation point (8 min) were 2% and 1%, respectively, while the yield of cis-2-hexen-1-ol reached 80%. The appearance of trans-2-hexen-1-ol and 1-hexanol before the semi-hydrogenation point confirmed the occurrence of Paths 1′ and 3 (Figure 7). After passing the semi-hydrogenation point, a portion of the accumulated cis-2-hexen-1-ol was also converted to trans-2-hexen-1-ol (Path 4), which was eventually reduced to 1-hexanol (Path 2′ in Figure 7). The composition of the reaction mixture changed similarly on the rest Pd/ZnO catalysts (Figure S2). The maximum cis-2-hexen-1-ol yields were determined to be 78% at 11 min for Pd/ZnO, 76% at 11 min for Pd-Pec3.5/ZnO, and 77% at 20 min for Pd-Pec8.1/ZnO. These data confirmed that Pd-Pec/ZnO catalysts with lower pectin content (1.8% and 3.5 wt) exhibited slightly higher activity than Pd/ZnO, whereas Pd-Pec8.1/ZnO demonstrated the lowest activity. The selectivity-versus-conversion curves (Figure 8d) showed that Pd/ZnO (97%) and Pd-Pec1.8/ZnO (96%) exhibited higher selectivity toward cis-2-hexen-1-ol compared to Pd-Pec3.5/ZnO (89%) and Pd-Pec8.1/ZnO (89%).

**Figure 9:**
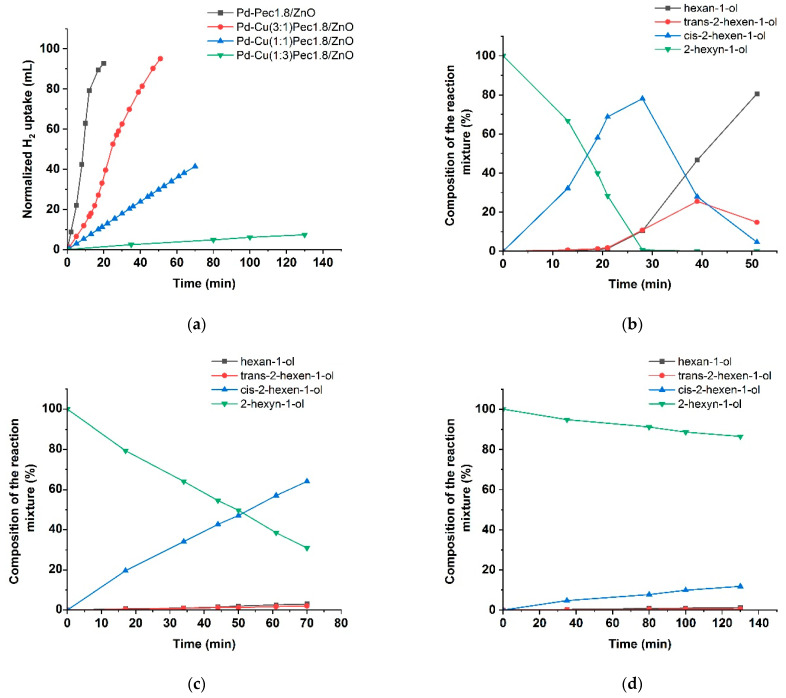
Hydrogenation of 2-hexyn-1-ol on PdCu-Pec1.8/ZnO catalysts: hydrogen uptake (**a**); changes in the composition of the reaction mixture in presence of PdCu(3:1)-Pec1.8/ZnO (**b**), PdCu(1:1)-Pec1.8/ZnO (**c**) and PdCu(1:3)-Pec1.8/ZnO (**d**). Reaction conditions: 50 mg of catalyst, 0.25 mL of 2-hexyn-1-ol, 25 mL of ethanol, at 40 °C and 0.1 MPa.

Figure 9 presents the results of testing the bimetallic PdCu-Pec1.8/ZnO catalysts. The modification with copper was shown to be significantly affected the activity of the optimized Pd-Pec1.8/ZnO catalyst. Compared to the monometallic Pd-Pec1.8/ZnO catalyst, which reached the semi-hydrogenation point in 8 min, the bimetallic PdCu(3:1)–Pec1.8/ZnO catalyst required a significantly longer time of 24 min under identical conditions. The other bimetallic catalysts exhibited negligible activity. Hydrogen uptake reached 41 mL for PdCu(1:1)-Pec1.8/ZnO (after 70 min) and 7 mL for PdCu(1:3)-Pec1.8/ZnO (after 130 min) (Figure 9a). This altered behavior can be attributed to the incorporation of copper, which, according to the XPS data (Figure 6), appears to cover the Pd species and suppress their reduction to the catalytically active metallic state (Pd^0^). The composition of the reaction mixture over PdCu(3:1)-Pec1.8/ZnO changed similarly to that observed for the monometallic analog, but at a slower rate. The maximum yield of cis-2-hexen-1-ol before reaching the semi-hydrogenation point (24 min) was 69% at 72% substrate conversion (data at 21 min), corresponding to 96% selectivity toward the target product (Figure 9b). The yield of cis-2-hexen-1-ol on PdCu(1:1)-Pec1.8/ZnO reached 64% at 69% substrate conversion (70 min) (Figure 9c), whereas for PdCu(1:3)-Pec1.8/ZnO this value did not exceed 12%, even after 130 min of reaction (Figure 9d).

The hydrogenation rate and selectivity of the catalysts, calculated from hydrogen uptake and chromatographic analysis data, respectively, are summarized in Table 3.

Modification with a minimal amount of pectin (1.8%) led to a slight improvement in the activity of the Pd/ZnO catalyst, while maintaining similarly high selectivity toward cis-2-hexen-1-ol. The increased activity can be attributed to the stabilizing effect of pectin, which promoted a more uniform distribution of palladium particles on the support surface. However, further increasing the pectin content to ~8% resulted in decreased activity and selectivity. This effect is likely due to steric hindrance restricting substrate access to the active sites caused by excess polymer. The addition of copper caused a significant decrease in catalyst activity, which is attributed to its influence on the state and structural environment of the palladium active sites. At the same time, selectivity was not improved, and even decreased, for samples with higher copper content.

**Figure 10:**
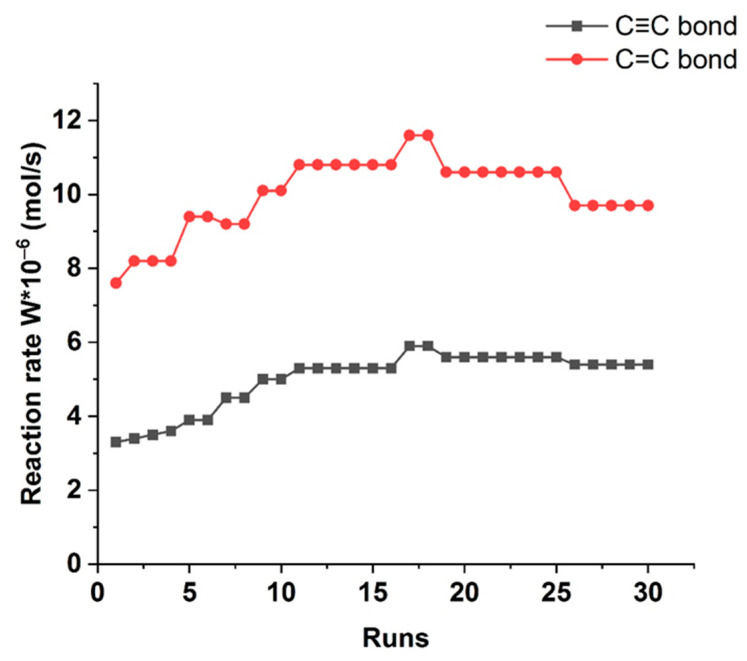
Reuse of Pd-Pec1.8/ZnO catalyst. Reaction conditions: 50 mg of catalyst, 0.25 mL of 2-hexyn-1-ol per cycle, 25 mL of ethanol, 40 °C and 0.1 MPa.

The most optimal Pd-Pec1.8/ZnO catalyst was successfully reused during 30 runs without loss in its activity (Figure 10). Moreover, after the first run, insignificant increase in catalytic efficiency was observed: the reaction rates of W_C≡C_ and W_C=C_ in the subsequent cycles reached 5.9 × 10^−6^ and 11.6 × 10^−6^ mol/s, respectively. This enhancement can be attributed to the more complete reduction of Pd^2+^ to catalytically active Pd^0^ during the initial reaction cycles [41]. Elemental analysis of the catalyst after 30 cycles showed that the palladium content did not decrease, but rather slightly increased from 0.96 wt% to 1.09 wt%. This indicates that Pd leaching is practically negligible. A minor increase in surface Pd concentration can be attributed to the reduction of PdO to metallic Pd during the reaction, resulting in surface enrichment of catalytically active Pd^0^ species. Atomic absorption spectroscopy (AAS) analysis of the reaction solution after 30 runs showed palladium levels comparable to those in the blank sample, indicating no measurable leaching and confirming the catalyst’s excellent stability.

The catalytic properties of Pd-Pec1.8/ZnO were compared to those of other reported Pd catalysts (Table 4). The values of selectivity to cis-olefinic alcohols (96%), the reaction rate in terms of TOF (0.7 s^−1^), and stability in terms of TON (29,000) were comparable to those of other advanced Pd-based catalysts supported on various materials. Moreover, after the first ten runs, the activity of the Pd-Pec1.8/ZnO catalyst increased, with the reaction rate in terms of TOF reaching up to 1.0 s^−1^, indicating excellent stability and potential for long-term application.

### 2.3. Perfomance of the Catalysts in Photocatalytic H_2_ Production

**Figure 11:**
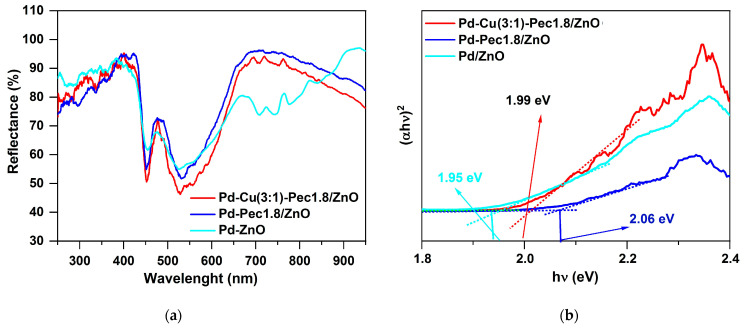
UV-vis reflectance spectra (**a**) and Tauc plots (**b**) for Pd/ZnO, Pd-Pec1.8/ZnO and Pd-Cu(3:1)-Pec1.8/ZnO.

Before conducting photocatalytic H_2_ evolution experiments, the optical properties of Pd/ZnO, Pd-Pec1.8/ZnO, and Pd-Cu(3:1)-Pec1.8/ZnO were evaluated using Ultraviolet–Visible diffuse reflectance spectroscopy (UV-vis DRS) method (Figure 11).

Figure 11a shows the UV–Vis diffuse reflectance spectra of the catalysts. Compared with the literature data for pristine ZnO, where the absorption edge is typically observed at ~380–400 nm [43], a noticeable red shift of the absorption edge to ~430–450 nm is observed for all palladium-containing samples. This shift may be attributed to the surface plasmon resonance (SPR) effect of metallic Pd nanoparticles, which extends the light absorption into the visible region [44]. The Pd/ZnO sample exhibits three distinct absorption bands (observed as reflectance minima) in the regions of approximately 400–500 nm, 500–600 nm, and 650–800 nm. These features likely originate from the superposition of interband transitions in ZnO, SPR of Pd, and possibly electronic transitions associated with palladium aggregates formed on the surface of ZnO [44]. Interestingly, in the pectin-modified samples (Pd-Pec1.8/ZnO and PdCu(3:1)-Pec1.8/ZnO), the broad absorption band in the 650–800 nm region is absent. This disappearance is probably due to stabilization role of pectin, preventing the aggregation of Pd nanoparticles on the surface of ZnO (Figure 4) and the formation of larger plasmon-active domains. Tauc plot analysis (Figure 11b) showed that the band gap values were 1.95, 1.99, and 2.06 eV for Pd/ZnO, PdCu(3:1)-Pec1.8/ZnO, and Pd-Pec1.8/ZnO, respectively. All values are significantly lower than that of pristine ZnO (~3.37 eV) [44], confirming the strong modifying effect of metal nanoparticles and Pec component on the electronic structure of ZnO.

**Figure 12:**
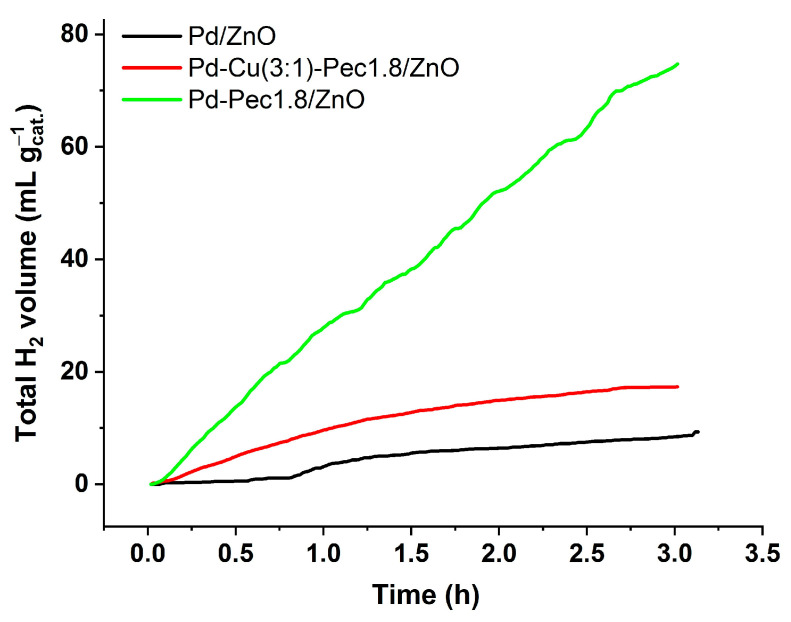
Photocatalytic H_2_ production efficiency of Pd/ZnO, Pd-Pec1.8/ZnO and Pd-Cu(3:1)-Pec1.8/ZnO. Reaction conditions: 70 mg of photocatalysts in 0.35 M Na_2_S and 0.25 M Na_2_SO_3_ aqueous solution, pH = 13, and light source—Xenon device (1000 W/m^2^).

Despite having the widest band gap, Pd–Pec1.8/ZnO demonstrated the highest photocatalytic activity for hydrogen evolution under visible-light irradiation. Conversely, Pd/ZnO, with the narrowest band gap, was the least active, while the bimetallic Pd–Cu(3:1)–Pec1.8/ZnO sample showed intermediate performance. The total amount of hydrogen produced within 3 h was 74.7, 17.3, and 9.3 mL/g_cat_ for Pd-Pec1.8/ZnO, PdCu(3:1)-Pec1.8/ZnO, and Pd/ZnO, respectively (Figure 12). Accordingly, the photocatalytic hydrogen evolution rate (PCHE) followed the order: Pd–Pec1.8/ZnO (1.11 mmol/(h·g_cat_)) ≫ PdCu(3:1)–Pec1.8/ZnO (0.26 mmol/(h·g_cat_)) > Pd/ZnO (0.14 mmol/(h·g_cat_)). The PCHE rate of Pd–Pec1.8/ZnO (1.11 mmol/(h·g_cat_)) is comparable to those of several advanced photocatalysts reported in the literature. For example, 5 wt% Pd–ZnS demonstrated a hydrogen evolution rate of 0.94 mmol/(h·g_cat_) under similar conditions (50 mg catalyst, Na_2_S/Na_2_SO_3_ as sacrificial agent, light intensity 100 mW cm^−2^) [45]. Likewise, Pd@TiO_2_CA achieved 0.76 mmol/(h·g_cat_) [41] and RGO–Cd_0_._60_Zn_0_._40_S–0.5%Mo reached 1.54 mmol/(h·g_cat_) [46], both under simulated solar light (1000 W m^−2^) with Na_2_S/Na_2_SO_3_ as sacrificial agents.

Pd–Pec1.8/ZnO and PdCu(3:1)–Pec1.8/ZnO demonstrated higher photocatalytic performance compared to Pd/ZnO. According to TEM studies (Figure 4), in both pectin-modified samples metal nanoparticles (Pd and Cu) with an average size of 3.4–3.6 nm were uniformly dispersed over the ZnO surface. In contrast, for Pd/ZnO, Pd nanoparticles (3.7 nm) tended to form larger aggregates with sizes up to 10–30 nm. This clearly indicates that the catalysts’ performance is primarily governed by their morphological and structural characteristics, rather than by differences in band gap values. A similar morphology-driven enhancement of photocatalytic activity was demonstrated by Wang et al. [47], who developed a homojunction photocatalyst based on CeO_2_ nanosheets with spatially separated oxidation and reduction phases located on opposite surfaces. The distinct crystalline facets of the smooth and rough sides enabled the efficient separation of photogenerated charge carriers. This morphological design resulted in a threefold increase in hydrogen evolution activity compared to pristine CeO_2_ nanosheets, and even outperformed Au-modified CeO_2_, despite the absence of noble metal co-catalysts. These findings highlight that structural and morphological features can exert a more significant impact on photocatalytic performance than conventional modifiers aimed at tuning the electronic and optical properties.

However, between the two pectin-modified samples, Pd–Pec1.8/ZnO exhibited superior activity compared to PdCu(3:1)–Pec1.8/ZnO, despite having a similar nanoparticle size and dispersion. XPS analysis provides insight into this difference (Figure 6). After H_2_ treatment, both catalysts contained Pd^0^ species, which are known to act as efficient electron traps [48], enhancing the spatial separation of photogenerated charge carriers via the ZnO–Pd Schottky junction. This suppresses charge recombination and promotes overall photocatalytic activity. In Pd–Pec1.8/ZnO, the high proportion of metallic Pd^0^ (~78%) provides a large number of active sites for electron trapping and catalytic hydrogen evolution. In contrast, PdCu(3:1)–Pec1.8/ZnO contains significantly less Pd^0^ (~22%), which already limits its efficiency. Furthermore, XPS analysis suggests that, in the bimetallic system, Pd nanoparticles may be partially or fully covered by a Cu-based shell, forming core–shell-like structures. This coverage can hinder the direct formation of a ZnO–Pd Schottky junction, which is crucial for efficient electron extraction and charge separation. As a result, the role of charge trapping may shift from Pd^0^ to Cu_2_O. The ability of Cu species (e.g., Cu_2_O) to facilitate charge separation is significantly inferior to that of metallic palladium (Pd^0^) [49]. Consequently, the photocatalytic efficiency of the bimetallic system remains lower, despite the presence of an additional component.

In summary, the superior activity of Pd–Pec1.8/ZnO can be attributed to (i) enhanced light absorption in the visible region, likely resulting from the plasmonic effect of Pd nanoparticles; (ii) more uniform dispersion of Pd nanoparticles, leading to improved charge separation and efficient utilization of active sites; and (iii) the absence of Pd–Cu interactions, which, in the bimetallic sample, hinder the reduction of Pd^2+^ to catalytically active Pd^0^, thereby limiting the formation of effective electron traps.

The long-term stability of the most active Pd–Pec1.8/ZnO catalyst was evaluated over three consecutive photocatalytic cycles (3 h each) under identical conditions without replacing the catalyst. The total hydrogen yield in the first run was 74.7 mL/g_cat_, while in the second and third runs it slightly decreased to 71.0 and 70.5 mL/g_cat_, respectively, corresponding to ~94.3% retention of the initial activity. These results confirm that the catalyst can be reused at least three times without significant loss of performance, demonstrating good stability and recyclability.

## 3. Materials and Methods

### 3.1. Chemicals and Materials

Zinc oxide (ZnO, pure grade), pectin (Pec, from apple, 50–75% esterification, Sigma-Aldrich, St. Louis, MO, USA), palladium (II) nitrate dihydrate (Pd(NO_3_)_2_ 2H_2_O, 40% Pd basis, Sigma-Aldrich, St. Louis, MO, USA), copper (II) nitrate trihydrate (Cu(NO_3_)_2_ 3H_2_O, 99.5%, Sigma-Aldrich, St. Louis, MO, USA), and ethanol (96.3%, Talgar Alcohol LLP, Talgar, Kazakhstan) were used without additional purification. The purity of 2-hexyn-1-ol (97%, Sigma-Aldrich, St. Louis, MO, USA) was confirmed by gas chromatography.

### 3.2. Synthesis of Pd-Polysaccharide/ZnO Catalysts

Monometallic Pd-Pec/ZnO catalysts were prepared by sequential adsorption of the polymer and palladium ions on zinc oxide as follows: A 10 mL of 0.01–0.05 M pectin solution (0.0185–0.0925 g of Pec in 10 mL of water) was added dropwise to the aqueous suspension of zinc oxide (1 g in 15 mL of water) under continuous stirring for 2 h. Then, a 5 mL of ~0.02 M Pd(NO_3_)_2_ aqueous solution was added dropwise, and the resulting mixture and stirred for 3 h. Furthermore, the resulting catalyst was kept in the mother liquor for 12–15 h, washed with distilled water and dried in air. The completeness of palladium immobilization was controlled using the photoelectric colorimetry method on a SF-2000 UV/Vis spectrophotometer (OKB Spectr, Saint Petersburg, Russia) using calibration curves at a wavelength of λ = 271 nm. The amount of added palladium salt and pectin was calculated to obtain Pd-Pec/ZnO catalysts with Pd content of 1 wt% and Pd to the polymer molar ratios of 1:1, 1:2, and 1:5. For comparison, similar unmodified Pd/ZnO catalyst was prepared using the same procedure except that the polymer was added.

### 3.3. Synthesis of PdCu-Pec/ZnO Catalysts

Bimetallic PdCu-Pec/ZnO catalysts were prepared using the same procedure as the monometallic palladium catalysts. Briefly, a 10 mL of 0.01 M pectin solution was added dropwise to the aqueous suspension of the ZnO (1 g in 15 mL of water). Afterward, metal ions were immobilized by successively adding 5 mL of Cu(NO_3_)_2_ and 5 mL of Pd(NO_3_)_2_ solutions to Pec/ZnO suspension. The concentrations of Pd(NO_3_)_2_ and Cu(NO_3_)_2_ solutions were varied within the range of 4.7 × 10^−3^–1.4 × 10^−2^ M and 7.9 × 10^−3^–2.4 × 10^−2^ M, respectively, to obtain 1% PdCu-Pec/ZnO catalysts with Pd:Cu molar ratios of 3:1, 1:1, and 1:3. The completeness of palladium and copper fixation was monitored using the photoelectric colorimetry method. The copper salt solutions were analyzed in the form of a copper–ammonia complex at a wavelength of λ = 620 nm.

### 3.4. Characterization of the Composites and Catalysts

Powder X-ray diffraction (XRD) patterns were obtained using a DRON-4-0.7 X-ray diffractometer (Bourevestnik, Saint Petersburg, Russia) using cobalt-monochromatized Co Kα radiation (λ = 0.179 nm). Ultraviolet–visible diffuse reflectance spectra were recorded using a T92+ UV-Vis spectrophotometer (PG instruments, Wibtoft, UK). The spectra were measured against BaSO_4_ at a wavelength range of 240–950 nm. The obtained diffuse reflectance data were converted to absorbance spectra using the Kubelka–Munk function. Band gap energies (Eg) were evaluated by Tauc plots of [F(R∞) E]^1/2^ versus photon energy E, where R∞ = R_Sample_/R_BaSO4_. Thermogravimetric analysis (TGA) of the catalyst was carried out in a nitrogen atmosphere (50 mL/min) at temperature range of 30–600 °C using a STA 449F5 analyzer (Netzsch, Selb, Germany) at a heating rate of 20 °C per minute. The elemental analysis was carried out using a JSM-6610LV (Jeol, Tokyo, Japan) scanning electron microscope with an EDX detector. Fourier transform infrared (FTIR) spectra of catalysts samples were obtained using a Nicolet iS5 (Thermo Fisher Scientific, Waltham, MA, USA) in the 4000–500 cm^−1^ region. Pellets for infrared analysis were obtained by grinding a mixture of a 1 mg sample with 100 mg dry KBr, followed by pressing the mixture into a mold. X-ray photoelectron spectra (XPS) of catalysts were recorded on an ESCALAB 250Xi X-ray and Ultraviolet Photoelectron spectrometer (Thermo Fisher Scientific, Waltham, MA, USA) with AlKα radiation (photon energy 1486.6 eV). Spectra were recorded in the constant pass energy mode at 50 eV for a survey spectrum and 20 eV for an element core level spectrum, using an XPS spot size of 650 μm. The total energy resolution of the experiment was approximately 0.3 eV. Investigations were carried out at room temperature in an ultrahigh vacuum of the order of 1 × 10^−9^ mbar. An ion-electronic charge compensation system was used to neutralize the sample charge. Transmission electron microscopy (TEM) micrographs and elemental mapping images were obtained using a Zeiss Libra 200FE transmission electron microscope (Carl Zeiss, Oberkochen, Germany) with an accelerating voltage of 100 kV.

### 3.5. Hydrogenation of 2-Hexyn-1-ol

The process was carried out in a thermostatically controlled long-necked glass flask reactor in an ethanol solution (25 mL) at 40 °C and atmospheric pressure of hydrogen, with intensive stirring (600–700 rounds per minute). A catalyst (50 mg) was pre-treated with hydrogen directly in the reactor at 40 °C for 30 min, and then 2-hexyn-1-ol was introduced in the reaction medium to perform the reaction. The substrate amount (0.25 mL) was taken based on the uptake of 100 mL of hydrogen. The reaction rate was calculated as the hydrogen consumption per unit of time. The amount of hydrogen uptake was determined by measuring the H_2_ volume in a gas storage burette connected to the reactor. The reaction products were analyzed by gas chromatography on a Chromos GC-1000 chromatograph (Chromos, Dzerzhinsk, Russia) with a flame ionization detector using a BP21 (FFAP) capillary column with a polar phase (PEG-modified with nitroterephthalate) of 50 m in length and a 0.32 mm inside diameter. The selectivity of the catalyst was calculated as the ratio of the target product to the sum of all reaction products at a fixed conversion. The reusability of the catalysts was evaluated by the hydrogenation rates in successive runs (0.25 mL, 2.23 mmol) using the same catalyst sample (50 mg) at 40 °C and atmospheric pressure of hydrogen. To assess palladium leaching, the reused catalyst was analyzed by EDX elemental analysis using a JSM-6610LV scanning electron microscope (JEOL, Tokyo, Japan) equipped with an EDX detector. Additionally, the reaction solution was analyzed for palladium content after 30 consecutive cycles using an MGA-1000 atomic absorption spectrometer (Lumex, St. Petersburg, Russia). For this analysis, 1 mL of the reaction mixture was diluted with 100 mL of 1% nitric acid. As a control, a blank solution containing 1 mL of ethanol in 100 mL of 1% nitric acid was also prepared and analyzed.

### 3.6. Photocatalytic H_2_ Evolution Experiments

Photocatalytic hydrogen production reactions took place in a homemade quartz reactor. An Asahi Spectra MAX-303 Xenon device (Asahi Spectra, Tokyo, Japan) was used as a light source during the photocatalytic hydrogen production measurements. The light source was adjusted to 1000 W/m^2^ via a light meter (HD 2302.0) in a manner that the source lighted an area of 3.5 × 3.5 cm of the base of the reactor in which the photocatalyst was fully located. Before the photocatalytic hydrogen production tests, the photocatalyst particles (70 mg) were suspended in aqueous solution of a 0.35 M Na_2_S and 0.25 M Na_2_SO_3_ mixture (pH = 13) of sacrificial reagent and allowed to settle down for 15 h. Immediately after the light irradiation, H_2_ generation took place, and the qualitative and quantitative characterization of the produced H_2_ was performed by connecting the photocatalytic reactor directly to an Agilent 6890 GC gas chromatograph (Agilent, Santa Clara, CA, USA). The stability of the catalyst was also evaluated under identical reaction conditions using the same catalyst batch (without replacement) over three consecutive photocatalytic cycles, each lasting 3 h.

## 4. Conclusions

This work is aimed to study the effect of the incorporation of pectin and copper into Pd/ZnO catalysts on their catalytic properties in liquid-phase hydrogenation of 2-hexyn-1-ol and photocatalytic hydrogen evolution. To this end, a series of monometallic Pd/ZnO catalysts with varying pectin contents (0 wt%, 1.8 wt%, 3.5 wt%, and 8.1 wt%), as well as bimetallic PdCu-Pec1.8/ZnO catalysts with different Pd to Cu mass ratios (3:1, 1:1, 1:3), were successfully synthesized via sequential adsorption of the polymer and metal ions on ZnO, followed by the comprehensive physicochemical characterization of the resulting catalysts. Modification of Pd/ZnO with a small amount of pectin (1.8 wt%) resulted in a slight increase in catalytic activity during hydrogenation while maintaining high selectivity toward cis-2-hexen-1-ol (96–97%). This enhancement is attributed to a more uniform distribution of Pd nanoparticles facilitated by the polymer. However, further increases in pectin content led to a decline in both activity and selectivity, likely due to steric hindrance imposed by excess polymer, which limited substrate access to the active sites. In contrast, the introduction of copper into Pd-Pec1.8/ZnO catalysts resulted in a significant decrease in both activity and selectivity. This effect was more pronounced with increasing Cu content and can be explained by the suppression of Pd reduction ability due to Pd–Cu interactions and coating of Pd active particles with Cu species, as confirmed by XPS analysis. The reduced proportion of metallic Pd likely compromised catalytic performance.

In photocatalytic hydrogen evolution, Pd-Pec1.8/ZnO outperformed both unmodified Pd/ZnO and bimetallic PdCu(3:1)-Pec1.8/ZnO, indicating a strong promoting effect from pectin modification. The observed trend in photocatalytic activity (Pd–Pec1.8/ZnO ≫ PdCu(3:1)–Pec1.8/ZnO > Pd/ZnO) suggests that pectin modification can favorably influence the electronic structure and increase the number of active sites on the catalyst surface. This enhancement is primarily attributed to the improved dispersion of Pd nanoparticles and the increased number of Pd–ZnO interfacial contacts, which facilitate the formation of Schottky junctions. These interfaces act as efficient electron traps, enhancing charge separation and suppressing recombination. In contrast, the co-introduction of copper appears to be detrimental due to unfavorable Pd–Cu interactions, which reduce the amount of metallic Pd^0^ and may result in partial coverage of Pd by Cu species, thereby limiting the formation of Pd–ZnO interfaces and decreasing charge trapping efficiency.

Overall, this study confirms that ZnO is an excellent support for palladium catalysts in hydrogenation reactions. While Pd/ZnO itself is already an effective catalyst, its performance can be further enhanced by modification with pectin, which improves the dispersion of Pd nanoparticles and increases catalytic efficiency in both liquid-phase hydrogenation and photocatalytic hydrogen evolution. The Pd-Pec1.8/ZnO catalyst demonstrates true bifunctional activity and holds promise for effective application in these two distinct processes. The demonstrated stability and high catalytic performance of the Pd–Pec1.8/ZnO catalyst over multiple cycles under both hydrogenation and photocatalytic conditions suggest its promising potential for scale-up. The catalyst’s robustness and reproducibility indicate feasibility for larger-scale applications, although further optimization of the synthesis protocols and reactor design will be necessary to fully realize its industrial implementation.

This work highlights the potential of polymer-modified Pd/ZnO systems and underscores the importance of precise control over catalyst composition to optimize performance.

## Figures and Tables

**Table 1:** The results of the assessment of pectin content in Pec/ZnO and Pd-Pec/ZnO composites.

m(Pec) in the Initial Solution, mg	m(Pec) in Solution after Sorption, mg	m(Pec) Adsorbed, mg	Adsorption Degree, %	Pec Content, %
Pec/ZnO				
18.5	0.0	18.5	100	1.8
37.0	2.2	34.8	94	3.4
92.5	37.8	54.7	59	5.0
Pd-Pec/ZnO				
18.5	0.0	18.5	100	1.8
37.0	0.0	37.0	100	3.5
92.5	3.4	89.1	96	8.1

**Table 2:** Results of assessing the degree of deposition of palladium and copper ions on ZnO and Pec/ZnO support materials.

Catalyst	Amount of Metal in Mother Liquor, mg				The Degree of Adsorption, %		Pd Content in a Catalyst, wt%			
	Before Sorption		After Sorption				PEC		EDX	
	Pd	Cu	Pd	Cu	Pd	Cu	Pd	Cu	Pd	Cu
Pd/ZnO	10.1	-	0.04	-	99.6	-	1.0	-	0.95	-
Pd-Pec1.8/ZnO	10.3	-	0.14	-	98.6	-	1.0	-	0.96	-
Pd-Pec3.5/ZnO	10.5	-	0.40	-	96.2	-	1.0	-	0.94	-
Pd-Pec8.1/ZnO	11.0	-	1.47	-	86.6	-	0.9	-	0.89	-
PdCu(3:1)-Pec1.8/ZnO	7.7	2.6	0.08	0.24	99.0	90.8	0.74	0.23	0.71	0.24
PdCu(1:1)-Pec1.8/ZnO	5.2	5.2	0	0.54	100	89.6	0.50	0.45	0.52	0.56
Pd-Cu(1:3)-Pec1.8/ZnO	2.6	7.7	0	0.32	100	95.8	0.25	0.72	0.17	0.81

**Table 3:** A comparison of catalytic properties of Pd/ZnO, Pd-Pec/ZnO and PdCu-Pec1.8/ZnO catalysts in hydrogenation of 2-hexyn-1-ol.

Catalyst	W × 10^−6^, mol/s		Selectivity to cis-2-hexen-1-ol, %	Conversion *, %
	C≡C	C=C		
Pd/ZnO	2.0	6.9	97	67
Pd-Pec1.8/ZnO	3.3	7.6	96	83
Pd-Pec3.5/ZnO	3.3	6.3	89	78
Pd-Pec8.1/ZnO	1.2	3.2	89	86
PdCu(3:1)-Pec1.8/ZnO	1.2	2.4	96	72
PdCu(1:1)-Pec1.8/ZnO	0.4	-	93	69
Pd-Cu(1:3)-Pec1.8/ZnO	0.1	-	87	13

*—The value of the substrate conversion used for the calculation of selectivity of the catalysts. Reaction conditions: 50 mg of catalyst, 0.25 mL of 2-hexyn-1-ol, 25 mL of ethanol, at 40 °C and 0.1 MPa.

**Table 4:** A comparison of catalytic properties of the Pd-Pec1.8/ZnO with those of other Pd catalysts.

Catalyst	Method of Preparation	Reaction Conditions	TOF, s^−1^	S _C=C_, %	TON	Ref.
Pd-Pec1.8/ZnO	Sequential adsorption: pectin (1.8 wt%) then Pd^2+^ on ZnO	50 mg cat., 0.25 mL 2-hexyn-1-ol, 25 mL C_2_H_5_OH, 40 °C, 0.1 MPa H_2_.	0.7	96	29,000	This study
Pd/ZnO-400	Strong electrostatic adsorption (SEA) of Pd^2+^ on ZnO, followed by H_2_ reduction at 400 °C (formation of intermetallic PdZn)	Continuous-flow reactor; 5 wt% 2-butyne-1,4-diol in water; 0.1 g catalyst; 80 °C; 2 MPa H_2_; contact time 29 g·h·mol^−1^	1.2	90	73,000	[6]
Pd/NC-ZIF-8	Wet impregnation of NC-ZIF-8 and PdCl_2_	4.9 mmol of 2-methyl-3-butyn-2-ol, 20 mL of water, 20 mg of catalyst, 35 °C, 0.5 MPa of H_2_, 1000 rpm of stirring rate.	0.4	95	1628	[42]
PdAg-HEC/ZnO	One-pot polysaccharide (hydroxyethyl cellulose, HEC) assisted deposition of Pd + Ag on ZnO	0.05 g catalyst, 0.09 mol/L 2-hexyn-1-ol in ethanol (25 mL), 40 °C, 0.1 MPa H_2_ (1 atm)	0.8	97	9700	[15]

## Data Availability

The data that support the findings of this study are available from the corresponding author upon reasonable request.

## Supplementary Materials

The following supporting information can be downloaded at: https://www.mdpi.com/article/10.3390/molecules30183806/s1, Figure S1: TEM microphotographs of Pd/ZnO (a), Pd-Pec1.8/ZnO (b), and PdCu(3:1)-Pec1.8/ZnO (c) at higher magnification; Figure S2: Changes in the composition of the reaction mixture during hydrogenation of 2-hexyn-1-ol on Pd/ZnO (a), Pd Pec3.5/ZnO (b), and Pd Pec8.1/ZnO (c).

## References

[1] Zhang L., Zhou M., Wang A., Zhang T. (2020). Selective Hydrogenation over Supported Metal Catalysts: From Nanoparticles to Single Atoms. Chem. Rev..

[2] Zhao X., Chang Y., Chen W.-J., Wu Q., Pan X., Chen K., Weng B. (2022). Recent Progress in Pd-Based Nanocatalysts for Selective Hydrogenation. ACS Omega.

[3] Chen X., Shi C., Liang C. (2021). Highly selective catalysts for the hydrogenation of alkynols: A review. Chin. J. Catal..

[4] Brito V.D., Achimón F., Dambolena J.S., Pizzolitto R.P., Zygadlo J.A. (2019). Trans-2-hexen-1-ol as a tool for the control of Fusarium verticillioides in stored maize grains. J. Stored Prod. Res..

[5] Zhang J., Hu W., Qian B., Li H., Sudduth B., Engelhard M., Zhang L., Hu J., Sun J., Zhang C. (2023). Tuning hydrogenation chemistry of Pd-based heterogeneous catalysts by introducing homogeneous-like ligands. Nat. Commun..

[6] Chen X., Shi C., Wang X.-B., Li W.-Y., Liang C. (2021). Intermetallic PdZn nanoparticles catalyze the continuous-flow hydrogenation of alkynols to *cis*-enols. Commun. Chem..

[7] Gholinejad M., Khosravi F., Afrasi M., Sansano J.M., Nájera C. (2021). Applications of bimetallic PdCu catalysts. Catal. Sci. Technol..

[8] Li J., Suo W., Huang Y., Chen M., Ma H., Liu C., Zhang H., Liang K., Dong Z. (2023). Mesoporous α-Al_2_O_3_-supported PdCu bimetallic nanoparticle catalyst for the selective semi-hydrogenation of alkynes. J. Colloid Interface Sci..

[9] Xu J., Guo X., Guan Y., Wu P. (2022). Influence of Pd deposition pH value on the performance of Pd-CuO/SiO_2_ catalyst for semi-hydrogenation of 2-methyl-3-butyn-2-ol (MBY). Chin. Chem. Lett..

[10] Oberhauser W., Frediani M., Dehcheshmeh I.M., Evangelisti C., Poggini L., Capozzoli L., Moghadam P.N. (2022). Selective Alkyne Semi-Hydrogenation by PdCu Nanoparticles Immobilized on Stereocomplexed Poly(lactic acid). ChemCatChem.

[11] Lu L., Zou S., Fang B. (2021). The Critical Impacts of Ligands on Heterogeneous Nanocatalysis: A Review. ACS Catal..

[12] Razzaque S., Hussain S.Z., Hussain I., Tan B. (2016). Design and Utility of Metal/Metal Oxide Nanoparticles Mediated by Thioether End-Functionalized Polymeric Ligands. Polymers.

[13] Jin L., Liu B., Duay S.S., He J. (2017). Engineering Surface Ligands of Noble Metal Nanocatalysts in Tuning the Product Selectivity. Catalysts.

[14] Akhmetova S., Zharmagambetova A., Talgatov E., Auyezkhanova A., Malgazhdarova M., Zhurinov M., Abilmagzhanov A., Jumekeyeva A., Kenzheyeva A. (2024). How the Chemical Properties of Polysaccharides Make It Possible to Design Various Types of Organic–Inorganic Composites for Catalytic Applications. Molecules.

[15] Zharmagambetova A.K., Talgatov E.T., Auyezkhanova A.S., Bukharbayeva F.U., Jumekeyeva A.I. (2023). Polysaccharide-Stabilized PdAg Nanocatalysts for Hydrogenation of 2-Hexyn-1-ol. Catalysts.

[16] Dagareh M.I., Hafeez H.Y., Mohammed J., Kafadi A.D.G., Suleiman A.B., Ndikilar C.E. (2024). Current trends and future perspectives on ZnO-based materials for robust and stable solar fuel (H_2_) generation. Chem. Phys. Impact.

[17] Bakranova D., Nagel D. (2023). ZnO for Photoelectrochemical Hydrogen Generation. Clean Technol..

[18] Park J.S., Kim B.J., Seo B.G., Han G.D., Park K.-H., Koo J., Park H.-D., Shim J.H. (2022). Hetero-structured palladium-coated zinc oxide photocatalysts for sustainable water treatment. J. Water Process Eng..

[19] Manzoor M.F., Ahmed E., Ahmad M., Ahmad I., Rana A.M., Ali A., Ghouri M.I., Manzoor M.S., Aziz M.T. (2020). Enhanced photocatalytic activity of hydrogen evolution through Cu incorporated ZnO nano composites. Mater. Sci. Semicond. Process..

[20] Zharmagambetova A.K., Talgatov E.T., Auyezkhanova A.S., Tumabayev N.Z., Bukharbayeva F.U. (2020). Effect of polyvinylpyrrolidone on the catalytic properties of Pd/γ-Fe_2_O_3_ in phenylacetylene hydrogenation. Reac. Kinet. Mech. Cat..

[21] Ahn S., Halake K., Lee J. (2017). Antioxidant and ion-induced gelation functions of pectins enabled by polyphenol conjugation. Int. J. Biol. Macromol..

[22] Minhas M.U., Ahmad M., Anwar J., Khan S. (2016). Synthesis and Characterization of Biodegradable Hydrogels for Oral Delivery of 5-Fluorouracil Targeted to Colon: Screening with Preliminary In Vivo Studies. Adv. Polym. Technol..

[23] Auyezkhanova A., Talgatov E., Akhmetova S., Kapysheva U., Zharmagambetova A. (2020). Synthesis and protective properties of pectin/montmorillonite composites against aspirin-induced enterocolitis. Periódico Tchê Química.

[24] Sumathra M., Govindaraj D., Jeyaraj M., Arfaj A.A., Munusamy M.A., Kumar S.S., Rajan M. (2017). Sustainable pectin fascinating hydroxyapatite nanocomposite scaffolds to enhance tissue regeneration. Sustain. Chem. Pharm..

[25] Thompson J.M. (2018). Infrared Spectroscopy.

[26] Urias-Orona V., Rascón-Chu A., Lizardi-Mendoza J., Carvajal-Millán E., Gardea A.A., Ramírez-Wong B. (2010). A Novel Pectin Material: Extraction, Characterization and Gelling Properties. Int. J. Mol. Sci..

[27] Shi L., Gunasekaran S. (2008). Preparation of Pectin–ZnO Nanocomposite. Nanoscale Res. Lett..

[28] Danková Z., Mockovčiaková A., Dolinská S. (2014). Influence of ultrasound irradiation on cadmium cations adsorption by montmorillonite. Desalination Water Treat..

[29] Wagner M., Pigliapochi R., Tullio V.D., Catalano J., Zumbulyadis N., Centeno S.A., Wang X., Chen K., Hung I., Gan Z. (2023). Multi-technique structural analysis of zinc carboxylates (soaps). Dalton Trans..

[30] Devi P.G., Velu A.S. (2016). Synthesis, structural and optical properties of pure ZnO and Co doped ZnO nanoparticles prepared by the co-precipitation method. J. Theor. Appl. Phys..

[31] Collins G., O’Dwyer C., Holmes J.D. (2015). Colloidal Palladium Nanoparticles versus Commercial Palladium Catalysts for Suzuki Cross Coupling reactions—The Influence of Surface Functionalization. NSTI Adv. Mater.—TechConnect Briefs.

[32] Sarac B., Karazehir T., Yüce E., Mühlbacher M., Sarac A.S., Eckert J. (2021). Porosity and thickness effect of Pd–Cu–Si metallic glasses on electrocatalytic hydrogen production and storage. Mater. Des..

[33] Sasaki H., Sakamoto K., Mori M., Sakamoto T. (2020). Synthesis of Ce_1−x_Pd_x_O_2−δ_ Solid Solution in Molten Nitrate. Catalysts.

[34] Talgatov E.T., Naizabayev A.A., Bukharbayeva F.U., Kenzheyeva A.M., Yersaiyn R., Auyezkhanova A.S., Akhmetova S.N., Zhizhin E.V., Brodskiy A.R. (2024). Pd Catalysts Supported on Mixed Iron and Titanium Oxides in Phenylacetylene Hydrogenation: Effect of TiO_2_ Content in Magnetic Support Material. Nanomaterials.

[35] Ghodselahi T., Vesaghi M.A., Shafiekhani A., Baghizadeh A., Lameii M. (2008). XPS study of the Cu@Cu_2_O core-shell nanoparticles. Appl. Surf. Sci..

[36] Du Z.-Y., Wang K., Xie Y.-M., Zhao Y., Qian Z.-X., Li S.-B., Zheng Q.-N., Tian J.-H., Rudnev A.V., Zhang Y.-J. (2024). In situ Raman reveals the critical role of Pd in electrocatalytic CO_2_ reduction to CH_4_ on Cu-based catalysts. J. Chem. Phys..

[37] Liu Q., Li Z., Zhou X., Xiao J., Han Z., Jiang X., Fu G., Tang Y. (2022). Cyanogel-Induced PdCu Alloy with Pd-Enriched Surface for Formic Acid Oxidation and Oxygen Reduction. Adv. Energy Sustain. Res..

[38] Wang T., Xu Y., Yang J., Ju X., Ding W., Zhu Y. (2019). Predictable Catalysis of Electron-Rich Palladium Catalyst toward Aldehydes Hydrogenation. ChemCatChem..

[39] Mierczyński P., Mierczyńska-Vasilev A., Maniukiewicz W., Vasilev K., Szynkowska-Jóźwik M. (2023). Novel Cu and Pd-Cu Catalysts Supported on Multi-Walled Carbon Nanotubes for Steam Reforming and Decomposition of Methanol. Catalysts.

[40] Raksha C.H., Yogeesh M.P., Shetty N.S. (2025). Recent advances in the synthesis of polymer supported catalysts: A review. Discov. Appl. Sci..

[41] Talgatov E.T., Naizabayev A.A., Kenzheyeva A.M., Myltykbayeva Z.K., Koca A., Bukharbayeva F.U., Akhmetova S.N., Yersaiyn R., Auyezkhanova A.S. (2024). Investigation of the Performances of TiO_2_ and Pd@TiO_2_ in Photocatalytic Hydrogen Evolution and Hydrogenation of Acetylenic Compounds for Application in Photocatalytic Transfer Hydrogenation. Catalysts.

[42] Shi J., Dou K., Xie D., Zhang F. (2024). Semi-hydrogenation of acetylenic alcohol to olefinic alcohol catalyzed by Pd nanoparticles embedded in nitrogen-enriched porous carbon derived from ZIF-8. Appl. Catal. O Open.

[43] Lu Y., Lin Y., Wang D., Wang L., Xie T., Jiang T. (2011). A high performance cobalt-doped ZnO visible light photocatalyst and its photogenerated charge transfer properties. Nano Res..

[44] Mbrouk O.A., Fawzy M., Elshafey H.M., Saif M., Hafez H., Mottaleb M.S.A.A. (2022). Green synthesized plasmonic Pd–ZnO nanomaterials for visible light-induced photobiogas production from industrial wastewater. Appl. Organomet Chem..

[45] Gaikwad A.P., Betty C.A., Jagannath, Kumar A., Sasikala R. (2018). Microflowers of Pd doped ZnS for visible light photocatalytic and photoelectrochemical applications. Mater. Sci. Semicond. Process..

[46] Akyüz D., Ozkaya A.R., Koca A. (2020). Photocatalytic and photoelectrochemical performances of Mo, Ni and Cu decorated metal chalcogenides. Mater. Sci. Semicond. Process..

[47] Wang X.-J., Yuan S.-S., Yang L., Dong Y., Chen Y.-M., Zhang W.-X., Chen C.-X., Zhang Q.-T., Ohno T. (2023). Spatially charge-separated 2D homojunction for photocatalytic hydrogen production. Rare Met..

[48] Li B., Wang R., Shao X., Shao L., Zhang B. (2017). Synergistically enhanced photocatalysis from plasmonics and a co-catalyst in Au@ZnO–Pd ternary core–shell nanostructures. Inorg. Chem. Front..

[49] Ramírez-Ortega D., Guerrero-Araque D., Próspero Acevedo-Peña P., Lartundo-Rojas L., Zanella R. (2020). Effect of Pd and Cu co-catalyst on the charge carrier trapping, recombination and transfer during photocatalytic hydrogen evolution over WO_3_–TiO_2_ heterojunction. J. Mater. Sci..

