# Supplementary material for: Effect of Pectin and Copper Modification on the Performance of Pd/ZnO Catalyst in Liquid-Phase Hydrogenation and Photocatalytic Hydrogen Evolution
Source: Molecules. 2025 Sep 18;30(18):3806. doi: 10.3390/molecules30183806 (PMC12472442; doi:10.3390/molecules30183806)
Supplement: Supplementary file 1 [file molecules-30-03806-s001.zip › molecules-3860732-supplementary.pdf]

Supplementary Information for:

Effect of Pectin and Copper Modification on the Performance of Pd/ZnO Catalyst in Liquid-Phase Hydrogenation and Photocatalytic Hydrogen Evolution

Alima M. Kenzheyeva [1,2,*], Alima K. Zharmagambetova [1], Eldar T. Talgatov [1,*], Aigul T. Zamanbekova [1], Aigul I. Jumekeyeva [1], Assemgul S. Auyezkhanova [1], Zhannur K. Myltykbayeva [3] and Atıf Koca [4]

[1] Laboratory of Organic Catalysis, D.V. Sokolsky Institute of Fuel, Catalysis, and Electrochemistry, Kunaev Str. 142, Almaty 050010, Kazakhstan
[2] Abai Kazakh National Pedagogical University, Almaty 050010, Kazakhstan
[3] Research Institute of New Chemical Technologies and Materials, Al-Farabi Kazakh National University, Almaty 050040, Kazakhstan
[4] Department of Chemical Engineering, Faculty of Engineering, Marmara University, Istanbul 34854, Türkiye
* Correspondence: a.kenzheeva@ifce.kz (A.M.K.); e.talgatov@ifce.kz (E.T.T.)

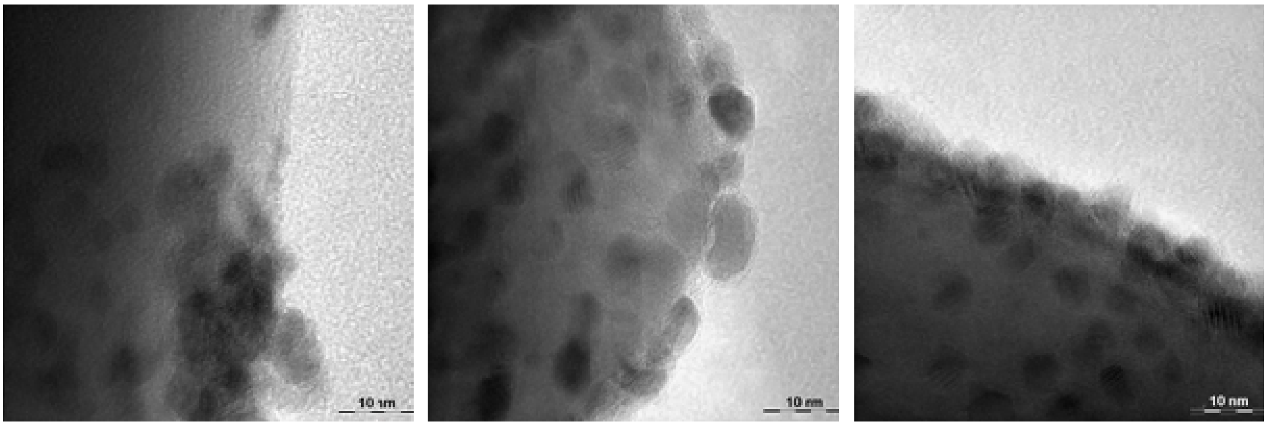

(a) (b) (c)

Figure S1. TEM microphotographs of Pd/ZnO (**a**), Pd-Pec1.8/ZnO (**b**), and PdCu(3:1)-Pec1.8/ZnO (**c**) at a higher magnification.

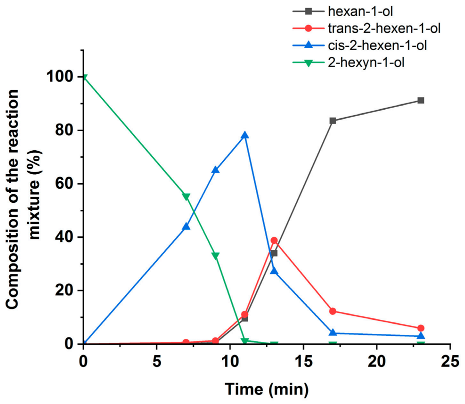

(a)

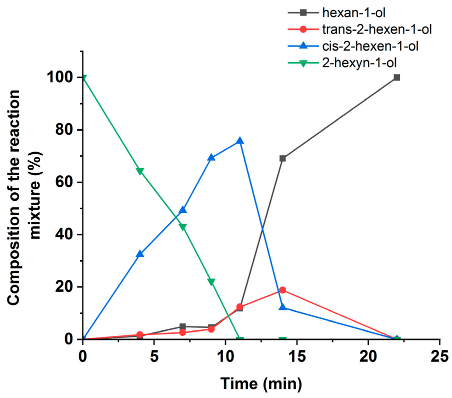

(b)

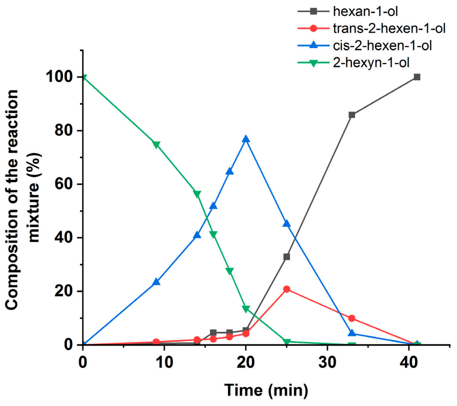

(c)

Figure S2. Changes in the composition of the reaction mixture during hydrogenation of 2-hexyn-1-ol on Pd/ZnO (**a**), Pd Pec3.5/ZnO (**b**), and Pd Pec8.1/ZnO (**c**). Reaction conditions: 50 mg of catalyst, 0.25 mL of 2-hexyn-1-ol, 25 mL of ethanol, at 40 °C and 0.1 MPa.
